# First note of QTL mapping of low vigor traits using the updated F2 ‘Koroneiki’ linkage map of olive

**DOI:** 10.3389/fpls.2025.1519402

**Published:** 2025-01-31

**Authors:** Irene Granata, Aparna S. Balan, Claudio Di Vaio, Antonino Ioppolo, Tiziano Caruso, Silvia Fretto, Jubina Benny, Antonio Giovino, Daniel James Sargent, Francesco Paolo Marra, Annalisa Marchese

**Affiliations:** ^1^ Department of Agricultural, Food and Forestry Sciences (SAAF), University of Palermo, Palermo, Italy; ^2^ Scitel Scientific Solutions Private Limited, Kayamkulam, Kerala, India; ^3^ Department of Agricultural Sciences, University of Naples Federico II, Portici, Italy; ^4^ Council for Agricultural Research and Economics (CREA)—Research Centre for Plant Protection and Certification (CREA-DC), Palermo, Italy; ^5^ Department of Plant Genetics, National Institute of Agricultural Botany (NIAB), Cambridge, United Kingdom

**Keywords:** *Olea europaea*, QTL linkage map, F2 progeny, plant height, low-vigor, brachitic dwarf phenotype

## Abstract

The olive tree (*Olea europaea* L.), which characterizes the agriculture of the Mediterranean basin, faces challenges adapting to high-density orchards and mechanized cultivation. This study addresses a key issue: controlling tree size to enhance efficiency and manageability in olive cultivation. Utilizing genetic mapping methods, we have identified significant Quantitative Trait Loci (QTL) and candidate genes associated with low-vigor traits in olive trees. Our research on the ‘Koroneiki’ F2 progeny, which exhibits low vigor traits but remains underutilized in breeding programs, has pinpointed a QTL linked to trunk basal diameter—a trait correlated with plant height based on morphological measurements. Results underscore a strong genetic control of these traits, with a consistent correlation observed over time. We identified two candidate genes — Acid Phosphatase 1, Shikimate O-hydroxycinnamoyltransferase, and a SNP Marker likely associated with Calcium Responsive Proteins — each potentially interacting with plant hormones to influence growth. Controlling olive tree size presents several challenges, including the genetic complexity of polygenic traits like size and vigor, and limited rootstock options. By integrating reference genomes with our genetic analysis, we offer a conceptual advancement that could substantially accelerate breeding timelines compared to traditional approaches. Although genome editing is still a future possibility due to the complexity of olive genetics and the species’ recalcitrance to transformation, our study lays a foundational understanding to guide future breeding programs. By targeting the identified candidate genes, this research represents a pivotal step toward selecting new low-vigor genotypes and rootstocks, contributing to innovations in olive cultivation.

## 1 Introduction

The olive (*Olea europaea* L.) is one of the most ancient and traditional fruit trees in the Mediterranean basin, and the primary oil source in southern Europe. Belonging to the Oleaceae family, this species includes both cultivated (var. *europaea*) and wild (var. *sylvestris*) olives, with a diploid genetic structure (2n = 2x = 46), although many authors have described polyploidy (Palomo-Ríos et al., 2021), and its genome size is ca. 1.5 Gb (Loureiro et al., 2007) with a ~31% of the whole genome composed of tandem-repetitive sequences (Barghini et al., 2014).

Over the past two decades, the global olive harvesting area has expanded from 8.3 to 10.3 million hectares, resulting in a production increase from 16 to 23 million tons per annum (FAOSTAT, 2023). This growth can be attributed to several factors, including the growing recognition of the nutritional benefits of olive-derived products, and the establishment of high-density olive orchards. Increasing tree density in orchards can result in higher yields and reduced production costs through mechanized harvesting and pruning (Tous et al., 2010; Marino et al., 2017; Massenti et al., 2024) but traditional tree vigor is often unsuitable for intensive orchards. Plant size control is therefore necessary. One of the most challenging topics in agriculture is the variability of the “habitus” and architectural traits of fruit trees, particularly the control of plant height and vigor. Studies conducted for the genetic improvement of the olive tree by the Department of Agricultural, Food and Forestry Sciences (SAAF) of University of Palermo on the germplasm of Sicilian olive trees, have allowed the identification of some local accessions such as KALAT with promising agronomic characteristics suitable for mechanization (Marino et al., 2017; Massenti et al., 2022). Additionally, some extremely low vigor F2 genotypes have been derived from controlled self-fertilization of the self-compatible cv. Koroneiki (Marchese et al., 2016a, b).

For many fruit species the control of plant height has been obtained using rootstocks, and years of analysis and experimentation have led to the selection of clones and rootstocks able to contain the tree size in citrus (e.g. Hayat et al., 2022; apple (e.g. Atkinson and Else, 2001; Costes and García-Villanueva, 2007), peach, and cherry (Basile and DeJong, 2018). Olive trees are traditionally propagated by self-rooting; indeed, grafting is used only in exceptional cases compared to other fruit tree species (León et al., 2020) such as for difficult-to-root cultivars or to overcome biotic disease and abiotic stress. However, also for the olive tree, controlling the size of the crown, plant height, and vigor has been a subject of intense research over time (e.g. Rallo et al., 2008a, b; Hammami et al., 2011; Rallo et al., 2016; Marino et al., 2017; Massenti et al., 2022 and Massenti et al., 2024). However, the availability of rootstocks is limited, and their effectiveness has been the subject of research (Baldoni and Fontanazza, 1990; Pannelli et al., 2002; Romero et al., 2014; Rugini et al., 2016). Despite the use of cultivars ‘Sauri’, ‘Mahasan’, and ‘Barnea’ for this purpose, a decade-long trial did not produce substantial outcomes (Baldoni and Belaj, 2009). Currently, only a limited number of cultivars have been identified by clonal selection as suitable for super-high-density orchards, including ‘FS-17^®^’/’Favolosa’ (patented CNR 1165nv), obtained in 1993 by free pollination of ‘Frantoio’ (Fontanazza et al., 1998; Sonnoli, 2001; Lodolini et al., 2023), ‘Arbequina IRTA-i 18^®^’, ‘Arbosana i 43’, and ‘Koroneiki i 38’ (Tous et al., 1999; Marra et al., 2016; Marchese et al., 2016a; Marino et al., 2017, b). More recently some new cultivars have been released from breeding programs, such as ‘Chiquitita’ derived from a cross between ‘Picual’ (♀) and ‘Arbequina’ (♂) (Rallo et al., 2008a, b; Rallo et al., 2016), ‘Lecciana’ obtained by a controlled cross between cv. Arbosana (♀) × cv. Leccino (♂) as part of an international research agreement between Agromillora Iberia S.L.U. and University of Bari (Camposeo et al., 2021), ‘Oliana^®^’ derived from ‘Arbequina’ × ‘Arbosana’, ‘Coriana^®^’ and ‘Olidia^®^’ both obtained from the cross ‘Arbosana’ × ‘Koroneiki’ (Camposeo, 2017).

Genetic control of plant height is very complex. Studies on mutants of *Arabidopsis thaliana* and *Oriza sativa*, two herbaceous model plants, led to the identification of some genes involved in the regulation of plant height, including for example, the *GAI-GIBERELLIN INSENSITIVE* gene, involved in the response pathway of gibberellic acid (Gottlieb, 1986; Peng et al., 1997; Busov et al., 2008); the *BUD1* gene, encoding *MAP KINASE KINASE7*, which negatively regulates the polar transport of auxin (Dai et al., 2006; Jia et al., 2016); and *BUD2* encoding *S-Adenosynioxymethylene Decarboxylasi4* (SAMDC4) a functional enzyme for the synthesis of polyamines (Wang and Li, 2006, 2008; Cui et al., 2010). Genes encoding brassinosteroid (*BR*) compounds or involved in the signal pathway of brassinosteroids were also identified, e.g. *D2 Cytocrome P450*, reported by Hong et al. (2003) and gene *D61*, encoding for the *BR receptor* kinase type which controls the elongation of the internodes (Chory and Li, 1997; Li and Chory, 1997; Yamamuro et al., 2000; Wang and Li, 2006), and finally genes controlling ramification and apical dominance such as *MAX1*, encoding a member of the cytochrome *P450* family and producing carotenoid derivatives (Booker et al., 2005; Beltran and Stange, 2016). It has been demonstrated that plant growth in model plants such as rice, Arabidopsis and soybean is regulated by the interaction among brassinosteroids (BRs) and gibberellins (GAs) (reviewed in Castorina and Consonni, 2020) and complications arising from pleiotropic effects were described by Assefa et al. (2019). In several plant species, including the model dicots *Arabidopsis thaliana*, *Nicotiana benthamiana*, *Medicago truncatula*, and the forage crop alfalfa (*Medicago sativa*), even a slight reduction in the activity of the enzyme hydroxycinnamoyl CoA: shikimate hydroxycinnamoyl transferase (HCT) results in reduced lignin levels and severe dwarfing (Vanholme et al., 2013; Serrani-Yarce et al., 2021).

In recent decades, molecular markers have enabled the construction of genetic linkage maps for numerous fruit tree species, facilitating the identification of genes with monogenic control over qualitative characteristics and those involved in the expression of quantitative traits (Quantitative Trait Loci or QTL), allowing for the early selection of seedlings with desired genetic traits, a process known as Marker Assisted Selection (MAS). Linkage maps are available for several fruit trees, including apple (e.g. Hemmat et al., 1994; Cheng et al., 1996; Maliepaard et al., 1998; Liebhard et al., 2003; Fernández-Fernández et al., 2008; Antanaviciute et al., 2012; Liu et al., 2022), cherry (Olmstead et al., 2008; Clarke et al., 2009; Guajardo et al., 2015), peach (e.g. Chaparro et al., 1994; Baird et al., 1996; da Silva Linge et al., 2018; Shi et al., 2020; Kalluri et al., 2022) and almond (Sánchez-Pérez et al., 2007; Goonetilleke et al., 2018; Karci, 2023; Goonetilleke and Fernández i Martí, 2023).

In apple breeding, for instance, linkage mapping has led to the identification of markers defining the columnar habit (Co) and other QTLs associated with branching patterns, growth rates, internode lengths, and other characteristics (e. g. Liebhard et al., 2002; Kenis and Keulemans, 2005; Kenis et al., 2008; Segura et al., 2009). Additionally, major QTLs such as Dw1 and Dw2, which contribute to rootstock-induced dwarfing in apple scions, have been identified on linkage groups LG5 and LG11 of the dwarfing rootstock ‘M.9’ (Fazio et al., 2014; Foster et al., 2015). A third QTL associated with dwarfing has also been mapped on chromosome 13 in a previously unknown region (Harrison et al., 2016). Ongoing research is focused on characterizing the apple rootstock M432 progeny and identifying additional QTLs linked to the dwarfing phenotype, showing distorted segregation and lethality or sub-lethality (Antanaviciute et al., 2012).

In peach trees, dwarf phenotypes are primarily controlled by three main genes: Dw, Dw2, and Dw3 (e.g., Hansche, 1988; Hollender et al., 2016; Cantín et al., 2018). Another gene, the “N gene,” also affects growth, with nn homozygotes exhibiting short internodes and Nn heterozygotes being semi-dwarfs. The temperature-sensitive semi-dwarf (Tssd) locus influences internode length. Dw and Tssd have been mapped to the distal part of chromosome 6 and the proximal part of chromosome 3, respectively (Lu et al., 2016). Recent studies have identified mutations in the gibberellic acid receptor PpeGID1c linked to BD (Hollender et al., 2016). Research in an F2 population of the ‘Nectavantop’ peach cultivar identified a second SNP in the PpeGID1c gene, suggesting multiple mutations can cause the BD phenotype (Cantín et al., 2018). As a result, linkage mapping in olives has traditionally been conducted using the filial generation 1 (F1) with a two-way pseudo-testcross approach. The first F1 genetic linkage maps were primarily built using AFLP and RAPD markers, along with a smaller number of sequence-characterized markers, RFLPs, and microsatellites (SSRs) (De la Rosa et al., 2003; Wu et al., 2004; Aabidine et al., 2010; Khadari et al., 2010). These maps ranged from 879 cM to 3,823.2 cM in length and contained various densities of markers spanning the 23 olive chromosomes to varying degrees. Dominguez-Garcia et al. (2012) constructed a linkage map using diversity array technology (DArT) markers, which showed 257 markers covering 1,205.1 cM. There have been few mapping studies for dissecting agronomic traits in olives, and a limited number of markers have been identified for traits like fruit characteristics, flower traits, tree growth, and olive oil quality. Atienza et al. (2014) phenotyped olives for fruit and vigor traits, identifying QTLs related to economically important traits. Sadok et al. (2013) mapped QTLs for flowering and fruiting traits in olives. Hernández et al. (2017) identified QTLs controlling fatty acid composition in olive oil.

In 2016, the Department SAAF – University of Palermo conducted a deep study on the segregation of vegetative characteristics, concerning habit and vigor, of a progeny derived from the self-compatible cv. ‘Koroneiki’ through controlled selfing (Marchese et al., 2016b). The study identified 81 segregating genotypes out of 330 individuals, which were then used to develop the first F2 olive high-density sequence characterized SNP-based linkage map using genotyping by sequencing (Marchese et al., 2016a). Utilizing an F2 population in olive breeding offers a distinct advantage over an F1 mapping population by enhancing the ability to observe the segregation of quantitative traits, thereby allowing for the detection of transgressive segregation, which can lead to the identification of extreme phenotypes beyond the parental range. Next Generation Sequencing technology (NGS) has improved classical candidate-gene approaches, such as genetic mapping, by making them faster and extremely effective. This enables the investigation of variations, including SNPs, small indels, and structural variants which helps in the discovery of genes and alleles of interest.

Until now the F2 ‘Koroneiki’ map is the most highly saturated linkage map available for olive, spanning the expected 23 chromosomes associated with the base chromosome number for the olive species and covering a total genetic distance of 1,189.7 cM. The lack of a reference genome for the olive species in the period of the development of the map and the juvenility of the progeny did not allow the identifications of QTL and candidate genes related to vigor and plant height traits. Recently, the availability of the genomes of *Olea europaea* L. *sylvestris* (Unver et al., 2017) and of three olive cultivars *Olea europaea* L. subsp*. europaea* var. *europaea* − ‘Farga’ (Cruz et al., 2016); ‘Picual’ (Jiménez-Ruiz et al., 2020); ‘Arbequina’ (Rao et al., 2021) and ‘Leccino’ (Lv et al., 2024) − have provided new tools to assist in the selection and development of new varieties efficiently, enabling marker-assisted selection and possibly genomic selection (Muleo et al., 2016). The *sylvestris* (Unver et al., 2017) and the cv. Farga (Cruz et al., 2016) genomes obtained by NGS technologies consist of 1.31 G and 1.48 G with contig N50 values of 52.35 kb and 25.49 kb, respectively, and many scaffolds, not entirely anchored to the chromosomes (Rao et al., 2021). The ‘Picual’ olive genome was sequenced using Illumina HiSeq 2500 for short reads and PacBio RSII for long reads. This approach ensured high accuracy and coverage, resolving complex genomic regions; the genome size is 1.81 Gb (Jiménez-Ruiz et al., 2020). The genome of cv. Arbequina was obtained by PacBio third-generation sequencing and Oxford Nanopore third-generation sequencing (ONT) technology consist of 1.30 G with contig N50 of 4.67 Mb assembled into 23 chromosomes by a genome-wide chromosome conformation capture technique, Hi-C (Rao et al., 2021). Recently, Lv et al. (2024) successfully sequenced the genome of the cv. Leccino, known for its high-quality oil and resistance against *Xylella fastidiosa* (La Notte et al., 2024), using a comprehensive approach that combined PacBio HiFi reads, ONT ultra-long reads, and Hi-C data. The assembly is 1.28 Gb, encompassing 23 gapless chromosomes, and achieved high-quality 99.67% genome coverage. This comprehensive genome includes 70,138 protein-encoding genes and various repeats, enhancing the genetic basis, particularly on the genetics of olive oil production.

The availability of these reference genomes together with the morphological analyses of architectural traits of the ‘Koroneiki’ F2 progeny carried out for several years by the department SAAF-University of Palermo, provides an opportunity for the identification of putative QTL/s and candidate genes involved in low vigor traits. Therefore, our study aimed to map traits related to low vigor, using morphological data from the ‘Koroneiki’ F2 progeny collected over a decade, along with SNP sequences previously identified by Marchese et al. (2016a). These SNPs, previously unknown due to the lack of reference genomes, were aligned with available genomic information and used for QTL detection and candidate gene discovery. The resulting map is highly informative and valuable for the potential future genomic selection of these desirable traits in olive trees.

## 2 Material and methods

### 2.1 Phenotype evaluation

The morphological parameters were examined in 81 F2 segregating genotypes, originating from seedlings germinated in April 2011. A paternity test using SSR markers confirmed they originated from ‘Koroneiki’ selfing (Marchese et al., 2016a, b). After two years of cultivation, the plants were transplanted from pots to the open field, where they were spaced 2.00 x 1.50 meters and allowed to grow freely, under rainfed conditions. No pruning was conducted to ensure trees could grow naturally, preserving their inherent habit and vigor. Morphological traits were assessed at six-months, nine-months, and one-year intervals, and subsequently at 2 -, 3-, and 12-year-old stages during the winter season, following the methodology outlined by Hammami et al. (2011). Measurements included the plant height of the main axis and the basal diameter at a point 5 cm above the ground (**Supplementary Table S1**). Other morphological parameters evaluated included leaf dimensions (length and width), with 30 leaves collected from each tree, the number of nodes, the internode length of the main axis, up to the 3rd year, and of 3 to 5 lateral branchlets in the 12th year. The leaf area (cm²) and the H/L ratio were also calculated (**Supplementary Table S2**). The measurements, which followed the guidelines outlined in the “Manual for the Primary Characterization of Olive Cultivars of the Sicilian Germplasm” (Caruso et al., 2007), provided insights into plant growth patterns.

### 2.2 Drupe morphological analysis

Among the Koroneiki progeny, 14 genotypes have reached the adult stage producing fruits, but only five genotypes produced more than 50 fruits per plant, while the other genotypes are still in the juvenile stage. For each genotype, fifty fruits were collected and compared to those of the mother plant, as shown in **Supplementary Table S3**. The weight of each fruit and its respective pit was determined using an ordinary balance with a sensitivity of 0.01 g. The diameter and length (mm) of each fruit were measured using a digital caliper. Additionally, the pulp weight (g) was calculated by subtracting the pit weight from the total fruit weight. The pulp-to-seed weight ratio and the fruit length-to-diameter ratio were then calculated. The manual of Caruso et al. (2007) was used for performing the measurements. For the analysis of the drupes and pit measurements, the mean separation was conducted by Tukey’s honestly significant difference (p < 0.05) and different letters were assigned to indicate significant differences among genotypes and within each morphological parameter.

### 2.3 QTL mapping

The QTL analysis was conducted on the phenotypic data for the F2 ‘Koroneiki’ mapping progeny using MAPQTL 6.0 (Van Ooijen and Kyazma, 2009). The non-parametric Kruskal-Wallis test was employed to identify significant associations between individual markers and traits. This was followed by interval mapping with a step size of 1.0 cM, and the percentage of phenotypic variance explained, along with the associated LOD values, were computed. A LOD significance threshold of 3.2 was determined through a permutation test with 10,000 repetitions and was used to establish significance. The resulting LOD values were visualized using MapChart 2.3 chart function (Voorrips, 2002).

Once QTL was identified, the *Olea europaea* var. *sylvestris* genome (Unver et al., 2017) was downloaded from NCBI (GCF_002742605.1), and all the mapped markers were queried against the genome with mismatch 0 and multi-map 1 using STAR (https://github.com/alexdobin/STAR). We cross-mapped the results from STAR alignment and questioned against the *Olea europaea* var. *sylvestris* blast db. Those genes were treated as candidate genes and mapped. Mapchart (https://www.wur.nl/en/show/mapchart.htm) software was used to present linkage maps and QTLs. In addition, the L.G. 13 was blasted against the olive genomes of the cvs. ‘Farga’ (Cruz et al., 2016), ‘Leccino’ (Lv et al., 2024), and ‘Picual’ (Jiménez-Ruiz et al., 2020). Due to the short length of one SNP marker, belonging to the QTL, it was aligned with RNA sequencing data using BLAST. RNA sequencing is currently being conducted on dwarf and tall F2 Koroneiki progenies (data not shown, manuscript in preparation), leading to the identification of an mRNA corresponding to known olive genomes and the discovery of a potential candidate gene.

## 3 Results

### 3.1 Vigor trait correlation and morphologic analysis

**Figure 1:**
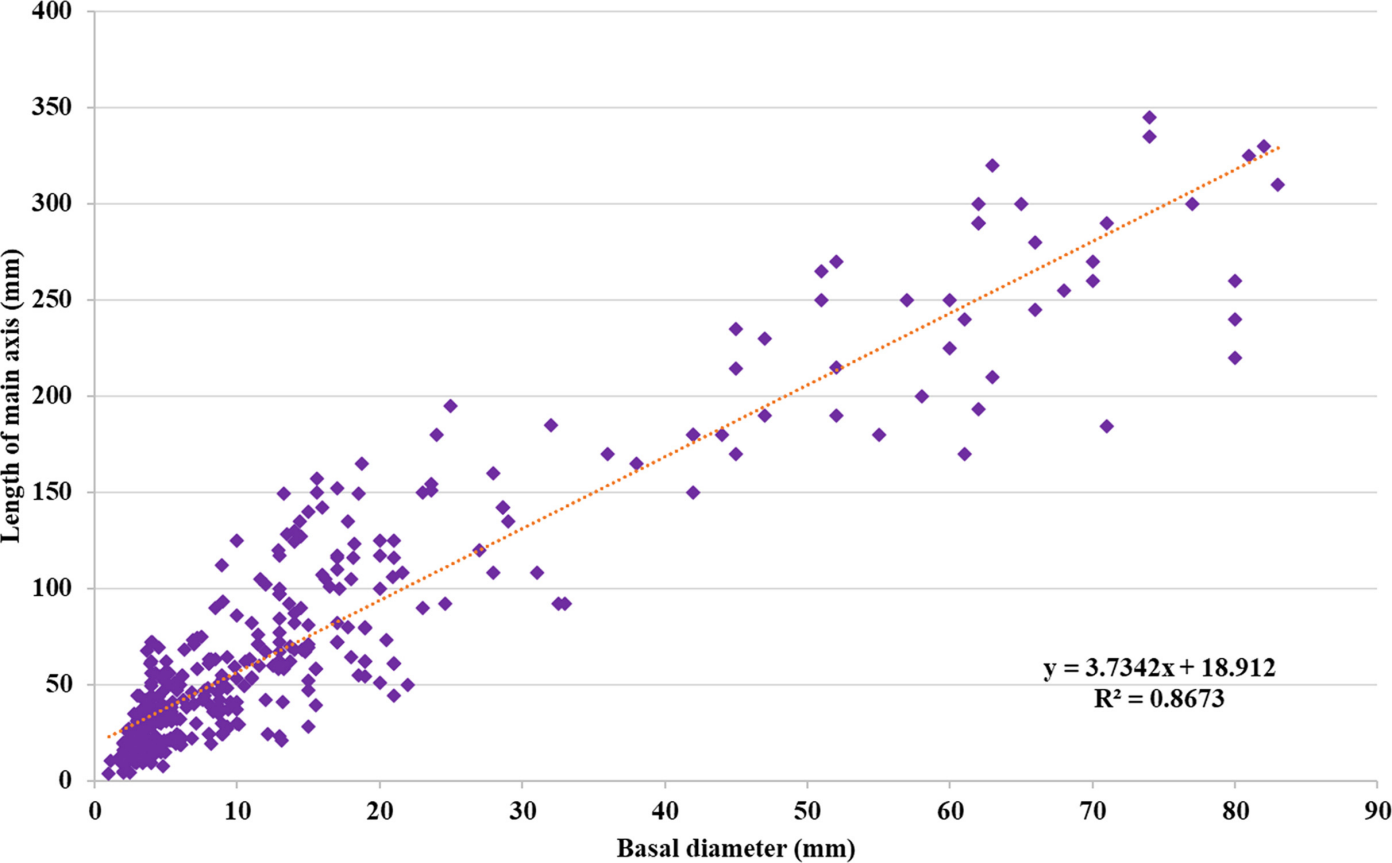
Regression between (d) basal diameter (mm) and (h) plant height (mm) of ‘Koroneiki’ F2 progeny.

To assess differences in growth patterns among the F2 ‘Koroneiki’ mapping progeny, plant height and basal diameter were recorded from 81 genotypes at 6 and 9 months during the first year, following the methods of Hammami et al. (2011), and when the plants were 2, 3, and 12 years old (**Supplementary Table S1**). The analysis of the data, based on observations of plant height and trunk diameter, showed a correlation index R^2^ equal to 0.87, as shown in **Figure 1**. By the third year, genotypes with a height greater than or equal to the ‘Koroneiki’ mother plant (150 cm) included 9 out of 81 (06, 19, 24, 26, 38, 49, 105, 237, 323), while 16 genotypes showed a basal diameter greater than or equal to 20 mm, similar to a ‘Koroneiki’ control plant. By the 12^th^ year, 35 genotypes had a height greater than 200 cm, and only 6 plants exceeded a height of 300 cm (06, 20, 24, 38, 49, 237). The basal diameter in 9 plants was lower than 20 mm, and these plants had a height lower than 100 cm (genotypes 22, 36, 67, 68, 148, 213, 246, 273, 325).

Regarding the number of nodes, the parental plant ‘Koroneiki’ presented an average number of nodes equal to 67 at the third year, while 43 out of 81 progenies exhibited an equal or greater number of nodes. A correlation of R^2^ equal 0.6 was found in the third year between the number of nodes and the plant height, while R^2^ was equal to 0.7 in the 12^th^ year. As for internode length, ‘Koroneiki’ presented an average of 2.2 cm at the third year, and only one genotype had an internode length around 2 cm; 19 out of 81 progenies presented internode lengths inferior to 1 cm, while the remaining fell in the range between 1 and 2 cm. In general, no correlation was found between internode length and plant height.

The morphological parameters of leaves (**Supplementary Table S2**) were measured in the third year, the standard ‘Koroneiki’ plant showed a leaf H/L ratio higher than 4 classifying them as elliptical-lanceolate, and only two genotypes, 51 and 126, exhibited the same shape. The remaining had a ratio below 4 and they can be classified as elliptical according to Caruso et al. (2007) classification manual. In the 12^th^ the leaf H/L ratio was again measured, and 10 genotypes showed a value greater than 4 (09, 19, 38, 51, 82, 216, 218, 246, 305, 313), giving them the same shape as the mother (elliptical-lanceolate), whereas all others had an elliptical shape. The leaf area was always smaller than that of the mother in the third year. While in the 12^th^ year, two genotypes 22 and 26 showed a leaf area greater than the mother (4.33 cm²).

### 3.2 Fruit analysis

The analysis of drupe characteristics (**Supplementary Table S3**) revealed that the drupe weight was significantly higher in genotype 49 (3.24 g), while genotype 24 (0.73 g) showed the lowest weight. Genotypes 38, 237, and 19 showed no significant differences compared to the ‘Koroneiki’ fruit weight (1.37 g). A similar trend was observed for pulp weight, with genotype 49 having the highest (2.65 g) and genotype 24 the lowest (0.56 g). The same trend was observed for pit weight, except that ‘38’ also exceeds the pit weight of the parental ‘Koroneiki’. Regarding the pulp-to-pit ratio, ‘Koroneiki’ exhibited the highest value (4.7), with no significant differences from ‘49’ and 38, while 24 (3.42) presented the lowest ratio. Concerning the fruit length-to-diameter ratio, ‘Koroneiki’ had the highest value (1.46), whereas 49 had the lowest (1.10). K19 not significantly differed from ‘Koroneiki’. The drupe length/diameter ratio is an important indicator of fruit shape. All genotypes of the K×K progeny were analyzed, including ‘Koroneiki’, displayed an elliptical shape, except for 49, which had spherical shape (Caruso et al., 2007). From a fruit morphology perspective, the pulp-to-pit ratio is a critical factor in assessing the overall oil yield of a genotype (Methamem et al., 2015). In this analysis, ‘Koroneiki’ exhibits the highest pulp-to-pit ratio among the genotypes of its progeny; however, the differences between K49 and K38 were insignificant. Therefore, this similarity suggests that these genotypes may possess comparable desirable characteristics regarding oil yield, making them attractive options for cultivation. Further investigations will be conducted in the future, including the assessment of their oil quality.

### 3.3 QTL mapping and candidate gene discovery

**Figure 2:**
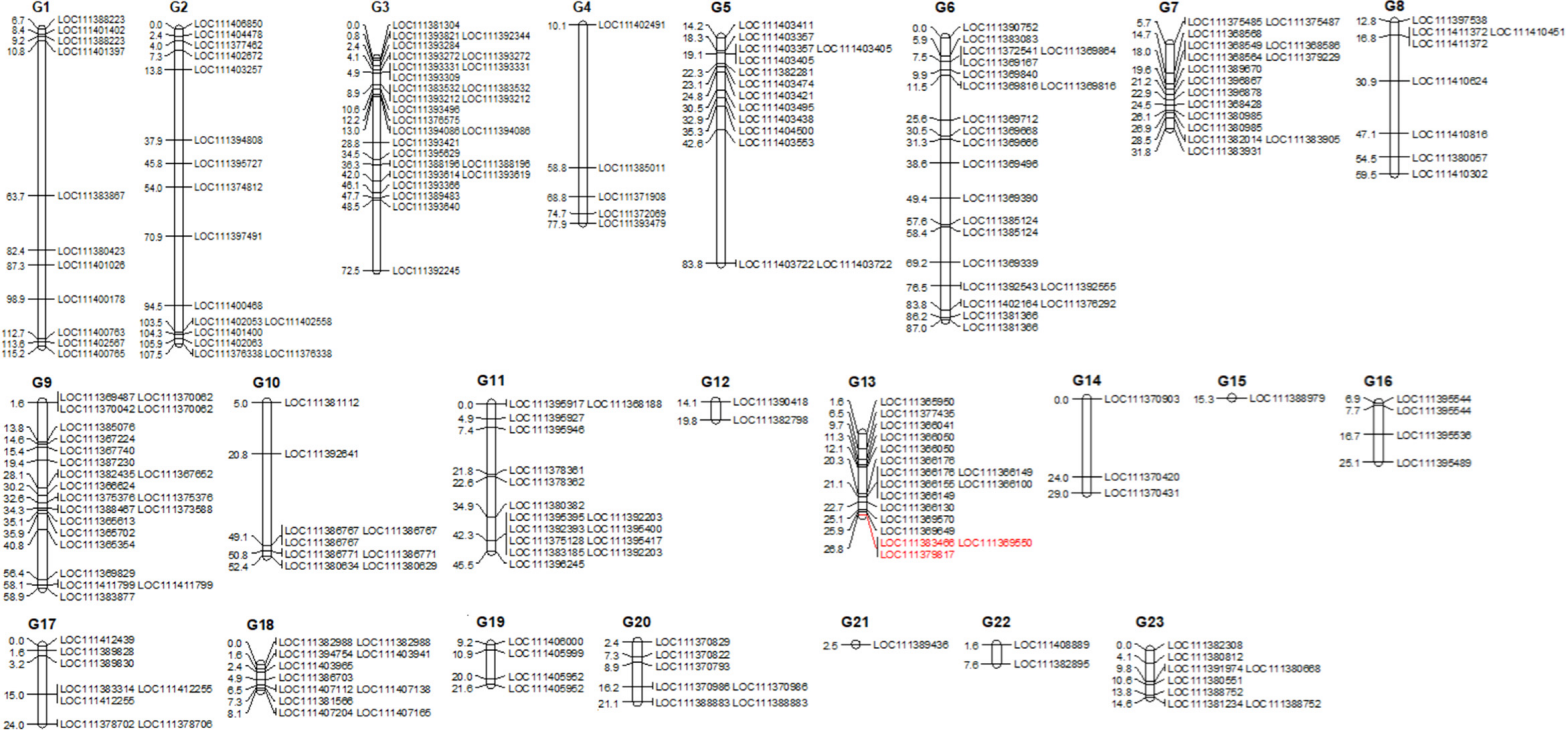
Updated Linkage Map of ‘Koroneiki’ F2 Progeny: This map displays selected SNP markers (Marchese et al., 2016a) with gene IDs, identified through BLAST analysis of the *Olea europaea* var. *sylvestris* genome. Candidate marker/gene names associated with plant basal diameter are highlighted in red. The QTL is represented by a bar alongside Linkage Group 13. A detailed map can be found in the **Supplementary Figure S1**.

**Figure 3:**
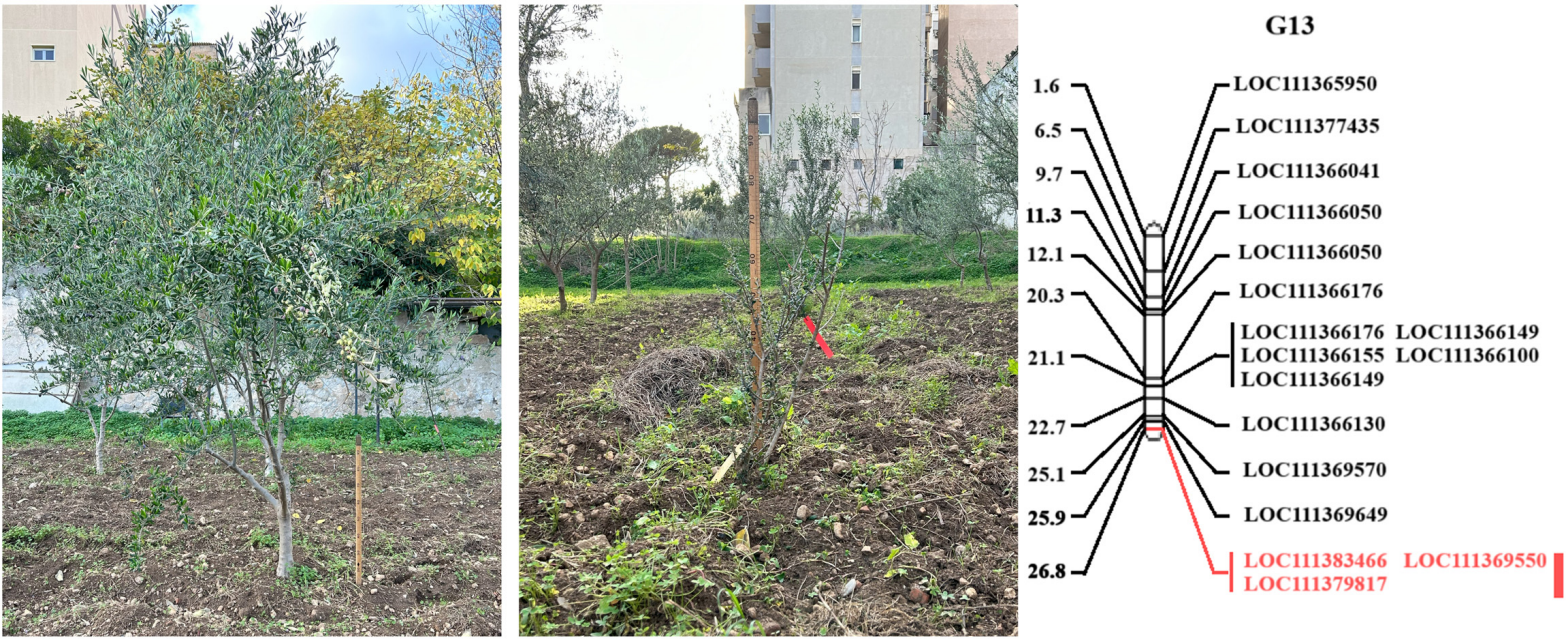
An example of height differences between ‘Koroneiki’ progenies and the L.G. 13 containing the QTL controlling plant basal diameter. Candidate genes - Acid phosphatase 1 (SNP: G13_33144; LOC111383466) and - Shikimate O-hydroxycinnamoyltransferase (SNP: G13_116650; LOC111379817) are highlighted in red; the red bar indicates the QTL region including the SNP Marker G13_12532 (likely associated with Calcium Responsive Proteins LOC 111369550).

Overall, of the 1,579 sequence characterized SNP markers mapped by Marchese et al. (2016a), 1359 SNPs (85.1%) corresponded to ‘Picual’, while 1137 (71.2%) matched to *Olea europaea* var. *sylvestris* (Unver et al., 2017) genomic regions (**Supplementary Table S4**; **Supplementary Figure S1**). For LG 13, where the major QTL was found, genes were also blasted against the cvs. ‘Farga’ (Cruz et al., 2016), Leccino’ (Lv et al., 2024), and ‘Picual’ (Jiménez-Ruiz et al., 2020) as shown in **Supplementary Table S5**. A major quantitative trait locus (QTL) associated with trunk basal diameter was discovered in linkage group (LG) 13, spanning a genetic distance from 13.62 cM to 17.84 cM, with a LOD score of 2.86, accounting for 19.4% of the phenotypic variation (**Figures 2**, **3** **;** **Supplementary Figure S1**). Our study identified two candidate genes: Acid Phosphatase 1 (SNP: G13_33144; LOC111383466) and - Shikimate O-hydroxycinnamoyltransferase (SNP: G13_116650; LOC111379817) and the SNP Marker G13_12532, likely corresponding to Calcium Responsive Proteins LOC 111369550 (**Figures 2**, **3** **;** **Supplementary Figure S1**).

## 4 Discussion

The analysis of the ‘Koroneiki’ progeny over twelve years provides significant insights into their growth patterns and phenotypic characteristics. According to Gottlieb (1986), plant architecture is determined by genetic factors that coordinate the growth and differentiation of metamers, which include the leaf, axillary bud, and internode. These genetic factors operate at the level of the whole plant, affecting traits such as shoot length and node number. Using methods from Hammami et al. (2011), plant height and basal diameter of the ‘Koroneiki’ progeny were systematically measured across 81 genotypes at various ages, revealing a strong correlation (R² = 0.87) between these traits and their developmental stages. By the third year, a subset of genotypes (9 out of 81) exceeded the parental plant’s height of 150 cm, while 16 achieved a basal diameter of at least 20 mm. This trend continued into the twelfth year, with 35 genotypes surpassing 200 cm in height, and 6 exceeding 300 cm. On the other hand, the remaining genotypes showed limited growth and were classified as “dwarf”. Of these, nine remained below 100 cm in height with a basal diameter of less than 20 mm, illustrating a range of growth dynamics within the progeny with extreme phenotype. While the standard ‘Koroneiki’ plant averaged 67 nodes, 43 progenies equaled or exceeded this count by the third year, and, generally, the progenies showing higher plant height presented a greater number of nodes. While there was not a clear correlation with the internode length. Leaf morphology predominantly was elliptical, though a minority displayed an elliptical-lanceolate shape as the mother plant. In the context of our study, the F2 progeny might be expressing juvenile traits that differ from the mature characteristics of the mother plant leaf. The correlation between the number of internodes (IN) and plant height (PH), but not with internode length, can be explained by the genetic and physiological factors influencing plant architecture. The number of nodes influences shoot length by determining the transition from vegetative to reproductive growth, as seen in the model plant pea where five loci govern node number (Murfet, 1977a, b). This suggests that node numbers are a key determinant of plant height, as they influence the plant’s overall structure and growth pattern. Assefa et al. (2019) using genome-wide association studies (GWAS) and genome-wide epistatic studies (GWES) recently identified multiple loci associated with the number of internodes and plant height, revealing a significant phenotypic correlation between IN and PH (r = 0.71), and the polygenic of many loci with small effect. Specifically, the Dt1 gene, that is an ortholog of Arabidopsis *Terminal Flower1*, encoding for phosphatidylethanolamine-binding ***proteins*** (Liu et al., 2010), located on chromosome 19, was highlighted as a significant locus influencing both plant height and internode number, suggesting also pleiotropic effects.

Different genetic factors involved in gibberellin metabolism influence internode length, which can vary independently of the number of nodes. For example, in pea plants, internode length is governed by five major loci, including the *Le* and *le* alleles, which are classic traits studied by Mendel, and the phenotypes produced by different combinations of alleles at these loci have been extensively studied (Reid et al., 1983). This indicates that internode length can be subject to specific genetic control that does not necessarily correlate with the overall plant height. Additionally, the coordination of growth and differentiation at the whole plant level, as described by Gottlieb (1986), suggests that while internode length may vary, the overall plant height and node number are more tightly regulated by genetic factors that ensure a consistent growth pattern. This could explain why our study found a correlation between plant height and node number but not with internode length.

The variation in the ‘Koroneiki’ progeny could result from the combined effects of multiple genes, each exerting a small influence on the phenotype. The genetic control of plant height is therefore very complex, with significant insights gained from studies on herbaceous model plants such as *Arabidopsis thaliana*, *Oryza sativa*, pea and soyabean. Key genes associated with height, particularly dwarf genes, are linked to plant hormones such as gibberellins (GA) and brassinosteroids (BR). These hormones interact within complex regulatory networks affecting plant stature (Li et al., 2024). Notable examples include the GAI gene, which responds to gibberellic acid (Peng et al., 1997; Busov et al., 2008), and the BUD1 gene, encoding MAP KINASE KINASE7, which negatively regulated auxin polar transport (Dai et al., 2006; Jia et al., 2016). The semidwarf1 allele in rice, a recessive mutation in the GA20 oxidase gene, was crucial during the Green Revolution to reduce plant size (Ashikari et al., 2002). Brassinosteroids are also critical in regulating plant height; deficiencies in these hormones cause dwarfism, while their overexpression can enhance height and branching Hong et al. (2003). Key genes involved in brassinosteroid signaling, such as the D2 Cytochrome P450 and the D61 gene, are essential for internode elongation (Li and Chory, 1997; Yamamuro et al., 2000; Wang and Li, 2006). Additionally, the phenylpropanoid pathway, particularly the activity of the hydroxycinnamoyl CoA: shikimate hydroxycinnamoyl transferase (HCT) enzyme, is linked to reduced lignin content and pronounced dwarfing (Vanholme et al., 2013; Dong and Lin, 2021; Serrani-Yarce et al., 2021).

In woody crops like apple trees, dwarfing genes Dw1 and Dw2 significantly influence height and trunk size. Recent advancements in genetic mapping and sequencing have enhanced the understanding of dwarfing mechanisms, particularly in rootstocks like ‘M9’. Dw1, located in linkage group LG5, and Dw2 in LG11, significantly influence key traits such as height and trunk cross-sectional area (Fazio et al., 2014; Foster et al., 2015). Furthermore, the mapping of minor effect QTLs to various linkage groups has deepened our understanding of dwarf traits in apples (Fazio et al., 2014; Foster et al., 2015). The discovery of specific alleles, involved in auxin transport genes (Zhang et al., 2015) and mobile mRNAs (Li et al., 2024) further elucidates the genetic interactions governing plant morphology. A chromosome-level genome assembly for ‘M9’, along with other rootstocks and apple cultivars, has revealed the importance of the Dw1 locus and the auxin response factor 3 (MdARF3) in dwarfing (Li et al., 2024).

Moreover, the apple showcases a dwarf phenotype known as “crinkle dwarf,” which is distinctly characterized by stunted growth and crinkled leaves. This phenotype is intricately linked to hybrid incompatibility, a genetic phenomenon that arises from the interaction of incompatible alleles and is regulated by lethal or semi-lethal genes, as reported by Mbulawa (2020). The crinkle dwarf trait was mapped to linkage group (LG) 8 in ‘McIntosh’ and LG2 in ‘M.1’ Mbulawa (2020). Research on genetic incompatibility in rice has shown that it can lead to hybrid necrosis, impacting growth and reproductive success (Calvo-Baltanás et al., 2021). This issue is especially pertinent when considering F2 progeny in allogamous and self-incompatible species, such as the olive tree. Our ‘Koroneiki’ F2 progeny stands out as a unique case, providing valuable insights into the concept of inbreeding depression, a phenomenon that has not been previously documented in the context of olive cultivation.

Recent studies by Castric et al. (2024) and Raimondeau et al. (2024) identified a hemizygous supergene at the *S*-locus in olive trees, crucial for sporophytic self-incompatibility. The GA2ox-S gene regulates gibberellins, hormones that influence plant growth. Raimondeau et al. (2024) screened nine olive genotypes derived from ‘Koroneiki’ self-pollination and found double null alleles of the GA2ox-S gene, indicating genomic alterations, though these plants are viable. In the future, we will focus on our F2 progeny to determine how many trees exhibit double null alleles and whether this condition alters gibberellin regulation, potentially leading to phenotypic changes like reduced vigor or semi-lethal conditions, explaining the mortality of many dwarf genotypes observed over the years.

Dwarf phenotypes in peach trees are influenced by three significant genes: Dw, Dw2, and Dw3 (e.g. Hansche, 1988; Chaparro et al., 1994; Hollender et al., 2016; Cantín et al., 2018), with the Dw gene linked to gibberellic acid receptor functionality - PpeGID1c gene (Hollender et al., 2016; Lu et al., 2016), mapped at the distal part of G6 (Lu et al., 2016; Hollender et al., 2016; Cantín et al., 2018). Dwarfing is also influenced by a fourth gene (N), where nn homozygotes have short internodes, while Nn heterozygotes are semi-dwarfs (Monet and Salesses, 1975). Additionally, the temperature-sensitive semi-dwarf (Tssd) locus plays a role in regulating internode length. Fine-mapping of the Tssd locus has identified a genomic region containing 69 predicted genes, indicating that multiple genes are involved in controlling this dwarfing phenotype (Lu et al., 2016).

Genetic linkage maps in olive trees have been constructed using molecular markers, leading to the identification of QTLs associated with important traits. Sadok et al. (2013) mapped QTLs related to flowering and fruiting, while Atienza et al. (2014) focused on traits like fruit size, weight, and oil content. Hernández et al. (2017) mapped QTLs controlling fatty acid composition in olive oil. The availability of reference genomes for olive, such as *Olea europaea* L. *sylvestris*, and cultivars like ‘Arbequina’, ‘Farga’, ‘Picual’, and ‘Leccino’, provides tools for selecting and developing new varieties, including marker-assisted selection and potential genomic selection.

In this study, we have successfully mapped a quantitative trait locus (QTL) for the first time, which is associated with the morphological traits of basal diameter and plant height, accounting for 19.4% of the phenotypic variance of the trait, indicating that the specific genetic variation linked to this QTL contributes significantly to the observable differences in these traits among individuals within a population. Interestingly, Atienza et al. (2014) previously identified a QTL for trunk diameter within the ‘Picual’ linkage group, derived from the DArT-SSR-based olive map Picual × Arbequina developed by Dominguez-Garcia et al. (2012). This QTL accounted for 16% of the total phenotypic variation, and the presence of the associated marker was linked to an increase in trunk diameter. Similarly, in apple, the Dw1 gene, controlling dwarfism and located on linkage group LG5, and Dw2 on LG11, have been found to significantly influence key traits such as height and trunk cross-sectional area (Fazio et al., 2014; Foster et al., 2015).

Our results from the phenotypic evaluation indicate that the two morphological parameters under study, plant height and basal diameter, were highly statistically significant throughout the observation period. Furthermore, a strong positive correlation was established between these variables. It is widely accepted that vigor traits should correlate with basal trunk diameter and plant height, as supported by Hammami et al. (2011) and Basile and DeJong (2018). León et al. (2020) also found a positive correlation between early measurements of plant height and trunk diameter in 43 wild olive genotypes. Numerous authors, including De la Rosa et al. (2006) and Hammami et al. (2011), suggest that plant age significantly influences growth habits, and that early characterization at the seedling stage, either before field planting or simultaneously, is the most effective tool for selecting vigor traits. In our study, we observed that the correlation between plant height and trunk diameter in the F2 ‘Koroneiki’ progeny has remained constant at 0.7% from the assessment of seedlings at 6 months to the present day, 12 years later, indicating a strong genetic control of the trait.

Our study identified three candidate genes within the QTL of Linkage Group 13. The first candidate gene, Acid Phosphatase 1 is involved in dephosphorylation, which may modulate hormone signaling pathways that impact cell expansion and growth, as noted by Anad and Srivastava (2012). Recent studies found that acid phosphatases, such as OsACP1, play a crucial role in managing phosphorus balance and signaling during phosphate stress, as highlighted by Deng et al. (2021). In rice, OsACP1 facilitates the recycling of phosphorus within cells, affecting its availability and hormone signaling, which in turn influences growth. Phosphatases like OsACP1 may interact with hormones such as auxin and ethylene, thereby affecting plant growth and adaptation to nutrient stress, as discussed by Li et al. (2011). Furthermore, transgenic plants expressing a phosphatase gene have been shown to increase growth, sugar content, and yield, as detailed in a patent by Versitech Limited, demonstrating a method for enhancing plant growth and yield by introducing phosphatases into plants. https://patentimages.storage.googleapis.com/83/22/8e/a03807f6b8fc72/US20130291224A1.pdf.

The second candidate gene discovered is Shikimate O-hydroxycinnamoyltransferase (Oe17g694580), crucial in the phenylpropanoid pathway, which is essential for lignin biosynthesis and the production of secondary metabolites (Tzin and Galili, 2010; Mir et al., 2015). In *Arabidopsis thaliana*, silencing of the hydroxycinnamoyl-CoA shikimate/quinate hydroxycinnamoyl transferase (HCT) gene (Hoffmann et al., 2004) leads to a significant reduction in plant growth due to a redirection of metabolic flux toward flavonoid production. The accumulation of flavonoids inhibits auxin transport, which is critical for growth and development (Besseau et al., 2007). Within the phenylpropanoid pathway, p-coumaroyl CoA serves as a pivotal junction, directing either toward flavonoid biosynthesis via chalcone synthase (CHS) or toward lignin biosynthesis through HCT. Modulating this pathway can significantly influence growth phenotypes, as evidenced by HCT-silenced plants where auxin transport was inhibited due to flavonoid accumulation (Besseau et al., 2007). This highlights the delicate balance between lignin and flavonoid biosynthesis and its impact on plant growth. Consequently, alterations in this pathway can affect plant structure and growth by influencing cell wall composition and hormone transport.

The identification of the third candidate gene was achieved by aligning the short SNP marker G13_12532 found within the QTL in L.G. 13 with RNA sequencing data from dwarf and tall F2 Koroneiki progenies (data not shown, manuscript in preparation). This alignment revealed an mRNA corresponding to known olive genomes, leading to the discovery of the potential candidate gene: Calcium Responsive Proteins (CRPs), involved in physiological processes and stress responses in plants. This CRP group includes calcium-dependent protein kinases (CDPKs), calmodulin (CaM), calmodulin-like proteins (CMLs), and calcineurin B-like proteins (CBLs). They mediate calcium signaling by binding calcium ions (Ca^2+^), which act as secondary messengers (Valmonte et al., 2014; La Verde et al., 2018). CRPs help interpret Ca^2+^ signals and activate pathways related to biotic and abiotic stress responses (Wei and Li, 2021). Moreover, CRPs are implicated in regulating plant growth by influencing cell elongation and division. Some studies suggest that CRPs interact with hormonal signaling pathways, including auxins, brassinosteroids, and abscisic acid, to coordinate physiological and metabolic responses, ultimately modulating plant growth in response to environmental factors (Wang et al., 2024).

The analysis of the Open Reading Frames of the G13_116650 and G13_33144 showed the presence of two synonyms substitutions due to transversion event not changing the protein functions while in the G13_12532 it was found a mutation at the intron level. At the expression level we know that there are some differences among dwarf vs tall (data not shown). The possible interaction between these genes and plant hormones such as auxin, gibberellin, ethylene, and brassinosteroids is likely to play a significant role in determining plant architecture and low vigor traits in the F2 ‘Koroneiki’ mapping progeny. To further investigate these connections, a transcriptomic study is currently underway using the F2 ‘Koroneiki’ genotypes (brachtic dwarf *vs.* tall) to explore the involved pathways and the potential roles of hormones. The candidate genes identified, including Acid Phosphatase 1, Shikimate O hydroxycinnamoyltransferase, and the SNP Marker G13_12532 (likely associated with Calcium Responsive Proteins), could facilitate a more targeted approach in olive breeding programs aimed at selecting low vigor genotypes and rootstocks.

## 5 Conclusion

Identifying QTLs and candidate genes linked to low vigor traits offers valuable insights for olive breeding programs, addressing the challenge of managing large olive trees in high-density orchards. The absence of olive rootstocks able to reduce tree size limits modern oliviculture efficiency. Marker Assisted Selection (MAS) can expedite the development of olive varieties with controlled growth, enabling breeders to select traits associated with low vigor for easier harvesting and maintenance. Traditional olive breeding is time-consuming, often taking decades to produce new cultivars. MAS can shorten this timeline by focusing on genetic markers linked to desired traits.

The ‘Koroneiki’ F2 progeny presents a unique opportunity to investigate genetic diversity related to dwarf and brachitic traits in olives. The “brachitic dwarf habit” may be influenced by transgressive segregation and/or hybrid necrosis (inbreeding repression the opposite of heterosis), a phenomenon not previously documented in the literature for the olive tree. As the first F2 progeny obtained in olives, this progeny may allow breeders to efficiently identify promising candidates through Marker Assisted Selection (MAS). While many olive genotypes are currently resistant to transformation, MAS can prepare the groundwork for future genome editing advancements. By identifying target genes for editing, MAS will enhance the precision of genome editing technologies, leading to cultivars tailored to modern agricultural needs. Together, these advanced breeding tools promise to create olive varieties that improve yield and management ease.

## Data Availability

The datasets presented in this study can be found in online repositories. The names of the repository/repositories and accession number(s) can be found in the article/**Supplementary Material**.

## Supplementary material

Supplementary Figure S1 Updated ‘Koroneiki’ F2 progeny linkage map showing the gene IDs of the SNPs markers (Marchese et al., 2016a) derived by blasting the genome of *Olea europaea* var. *sylvestris* and candidate marker/gene names related to plant basal diameter highlighted in red. QTL is indicated as bar running alongside the linkage group 13.

Supplementary Table S1 Height and Basal diameter measurements of the ‘Koroneiki’ F2 Progeny.

Supplementary Table S2 Morphologic parameters of leaves, internodes and nodes measured.

Supplementary Table S3 Fruits morphologic parameters.

Supplementary Table S4 BLAST Analysis of SNPs in 23 Linkage Groups of 'Koroneiki' F2 Progeny Compared with the Olive Genomes of the cv. Picual and *sylvestris*.

Supplementary Table S5 Comparative BLAST Analysis of SNPs in Linkage Group 13 Across Various Published Olive Genomes.
