# Supplementary figures and images for: First note of QTL mapping of low vigor traits using the updated F2 ‘Koroneiki’ linkage map of olive
Source: Front Plant Sci. 2025 Jan 31;16:1519402. doi: 10.3389/fpls.2025.1519402 (PMC11825337; doi:10.3389/fpls.2025.1519402)

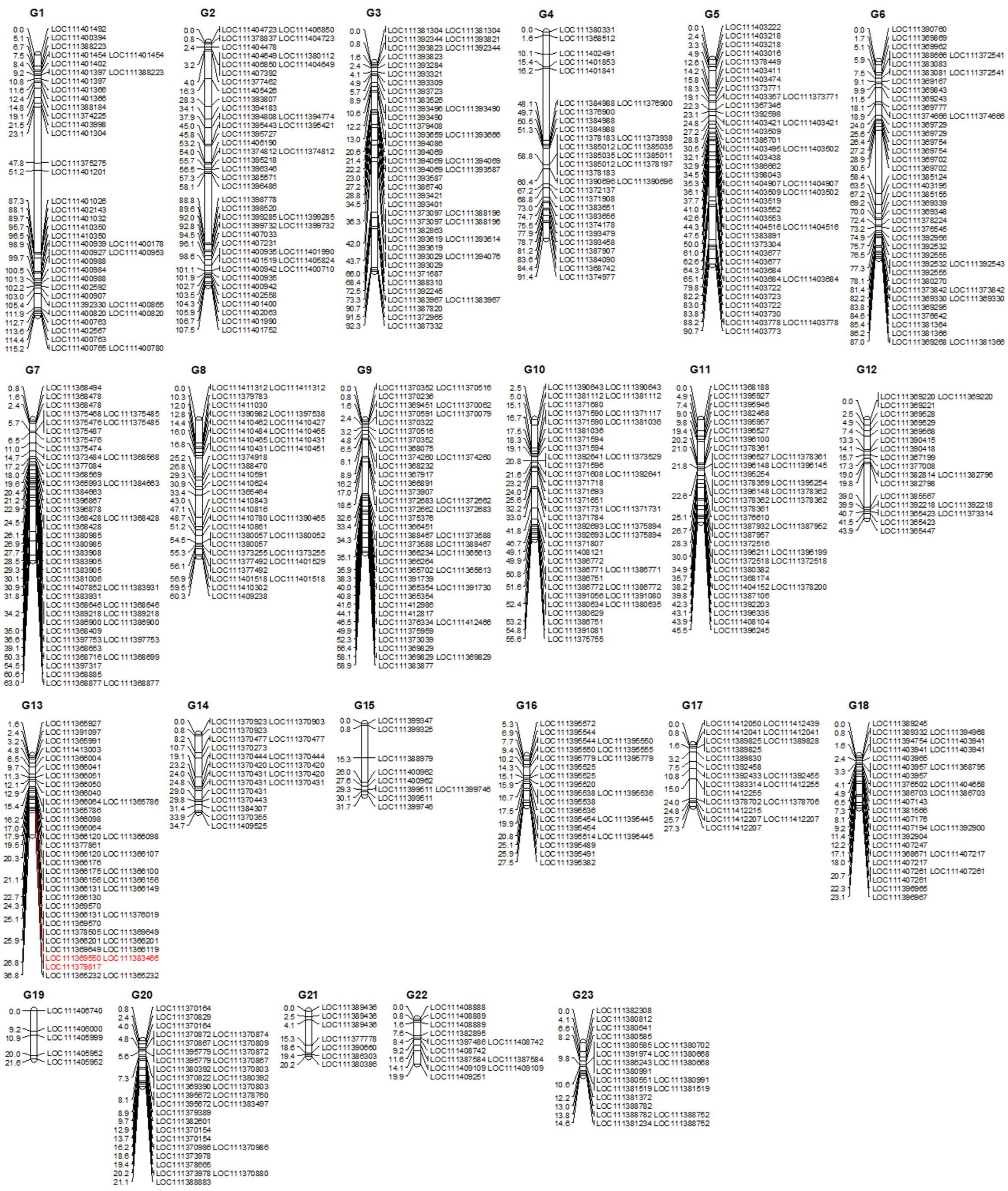

Supplement: Supplementary Figure S1 — Updated ‘Koroneiki’ F2 progeny linkage map showing the gene IDs of the SNPs markers (Marchese et al., 2016a) derived by blasting the genome of Olea europaea var. sylvestris and candidate marker/gene names related to plant basal diameter highlighted in red. QTL is indicated as bar running alongside the linkage group 13. [file Image1.tif]
